# Transection of the Vestigial Anterior Communicating Artery Limb during Microsurgical Clip Ligation of a Large, Ruptured Anterior Communicating Artery Trifurcation Aneurysm

**DOI:** 10.1055/a-2780-4233

**Published:** 2026-02-18

**Authors:** Romil Singh, Liam Cullen, Barnabas Obeng-Gyasi, Mokshal Porwal, Michael Meyer, Ethan Fitzgerald, Clementina Aiyudu, Paul Jeong, Evan Luther

**Affiliations:** 1 Department of Neurology, Allegheny General Hospital, Pittsburgh, Pennsylvania, United States; 2 School of Medicine, University of Pittsburgh, Pittsburgh, Pennsylvania, United States; 3 Department of Neurosurgery, Allegheny General Hospital, Pittsburgh, Pennsylvania, United States

**Keywords:** vestigial AComm, clipping, cerebrovascular, aneurysm

## Abstract

Unrecognized anatomic variants of the anterior communicating artery (AComm) complex can increase periprocedural risk during aneurysm treatment. A trifurcated AComm complex remains rare, with an incidence below 1%. Furthermore, vestigial AComm limbs may be difficult to appreciate on preoperative imaging. This video illustrates the intraoperative identification of a vestigial AComm limb and its transection to visualize and effectively clip a large, ruptured AComm trifurcation aneurysm. A middle-aged female presented with subarachnoid hemorrhage secondary to an 11-mm AComm aneurysm and was also found to have a small left anterior choroidal artery aneurysm. Preoperative imaging confirmed the presence of three A2 segments, but it was unclear if the left and right A1–A2 junctions communicated. Given her young age, aneurysm neck morphology, and the ability to treat both aneurysms through a single approach, microsurgical clip ligation was recommended. Following exposure of AComm complex, a small vestigial limb was identified, obscuring complete visualization of the aneurysm neck. Multiple fenestrated clip constructs were attempted but either occluded the A2 origin involved in the aneurysm neck or resulted in residual dome filling. The vestigial limb was transected, and the uninvolved A1–A2 junction was transposed to visualize the aneurysm neck, allowing for successful clipping fully. Intraoperative indocyanine green and postoperative angiography confirmed complete aneurysm occlusion and patency of all three A2s. Preoperative recognition of anatomic variants and their intraoperative confirmation can obviate complications in aneurysm surgery.

**Video 1** Intraoperative transection of a vestigial anterior communicating artery limb during microsurgical clip ligation of a ruptured AComm trifurcation aneurysm.


